# The Expression of *p53*, *CD44*, *Ki-67*, and *HER-2/neu* Markers in Gastric Cancer and Its Association with Histopathological Indicators: A Retrospective Study 

**DOI:** 10.31557/APJCP.2020.21.6.1607

**Published:** 2020-06

**Authors:** Mahsa Ahadi, Afshin Moradi, Leila Musavinejad, Abolfazl Movafagh, Arsham Moradi

**Affiliations:** 1 *Department of Pathology, School of Medicine, Shahid Beheshti University of Medical Sciences, Tehran, Iran. *; 2 *Department of Pathology, Shohada-e-Tajrish Hospital, Shahid Beheshti University of Medical Sciences, Tehran, Iran. *; 3 *Department of Medical Genetics, Schoolof Medicine, Shahid Behesti University of Medical Sciences, Tehran, Iran. *; 4 *Department of Biology, University of Toronto, Toronto, Canada. *

**Keywords:** p53, CD44, Ki-67, HER-2/neu, gastric cancer

## Abstract

**Background and objectives:**

Gastric cancer is known as one of the most common cancers and causes of deaths. Early and proper diagnosis is one of the most important things for treatment response. Therefore, this study aimed to determine the expression of* p53*, *CD44*, *Ki-67*, and *HER-2/neu* markers in the gastric cancer and its relationship with histopathological indicators.

**Methods:**

This is a descriptive-analytical study on 60 patientsts with cancer who underwent gastrectomy surgery in 2011-2016 in Shohadaye Tajrish Hospital. The participants were investigated for* p53*, *CD44, Ki-67*, and *HER-2/neu* markers’ staining plus demographic characteristics, rate of survival, and histopathological features of the tumors.

**Results:**

The mean age of the participants (44 males and 16 females) was 60.25±1.29 years. The patients’ survival rate was 23.82±1.56 months on average. The tumor size was reported as 6.09±2.61 cm and the major tumor type reported was intestinal type (n=40, 66.7%). The level of expression of* Ki-67* and* CD44 *makers was recorded as 33.75 and 24.50%, and *p53* and *HER-2/neu* immunostains were positive in 25 (41.7%) and 20 (33.3%) patients, respectively. The expression of* p53* and *CD44* markers had no significant relationship with the demographic characteristics, rate of survival, and histopathological features of the tumor of patients (all p>0.05). The expression of* p53* gene was associated with a lower rate of survival (p=0.014), while the expression of *HER-2/neu* was associated with higher probability of developing intestinal type of stomach adenocarcinoma (p=0.010) and ulcerative macroscopic view (p=0.034).

**Conclusion:**

This study illustrated that* p53* and *CD44* markers did not have any diagnostic value in predicting the biological behavior of gastric cancer. In fact, incidence of* p53* mmunoreactivity was associated with the lower rate of survival, and the expression of *HER-2/neu* was associated with higher probability of developing the intestinal type of stomach adenocarcinoma and ulcerative macroscopic view.

## Introduction

Based on the latest statistics of the cancer research center of the country, gastric cancer is the most common cancer among men and the third most common cancer among the Iranian women following breast cancer and colon cancer (Mohagheghi, 2004; Mohagheghi et al., 1998). Nevertheless, there is considerable geographical differences in this classification due to the effect of environmental and genetic factors (Morales, 1997). The main risk factor of distal gastric cancer includes infection with *H. pylori *and nutritional factors. On the other hand, gastroesophagael reflux disease (GERD) and obesity play a significant role in developing the cancer in the proximal part of the stomach (Correa 1992; Parsonnet et al., 1991; Trédaniel et al., 1997; DeVita et al., 2008 ).

At the molecular level, stomach tumor originates from several genetic variations, including oncogenes, tumor suppressor genes, and cell cycle regulators. However, their pathogenesis is still unknown. One of the most well-known of these genes is* p53* whose role in many cancers, including gastric cancer, has been well established (Smith et al., 2006; Jovanović et al., 2005). It is notable that mutation in* p53* is progressive, and patients with the mutated gene have a shorter lifespan compared to the patients, in which this gene has not been mutated (Milner and Watson, 1990). Investigation of the survival of patients across different studies suggested that overexpression of* p53* is associated with the histologic type of gastric cancers as well as the prognosis of the disease, and its role as a carcinogenic factor in the gastric cancer has been strongly recommended (Shun et al., 1997; Liu et al., 2001). Another gene is HER-2/neu and the gene related to it lies on chromosome 17 and produces a transmembrane glycoprotein, where this glycoprotein functions with a special tyrosine kinase (Kunz et al., 2012). In addition, some studies have associated overexpression of this gene with a worse prognosis, more aggressive behavior, and extensive growth (Al-Moundhri et al., 2005).

Ki-67 identifies a special proliferation antigen. It is expressed by the cells, which are in the terminal stages of G1, S, G2, and M phase, though it is not expressed in the cells in G0. It’s staining is usually nuclear or circum-nuclear. The extent of cellular proliferation, which is investigated through Ki-67 immunoreactivity, has been studied as a prognosis indicator in several malignant neoplasms and results showed its relationship with the size of tumor and its clinical course (Al Moundhri et al., 2005; Lindboe et al., 2003).

Considering the proof of the key role of mutation of some tumor suppressor genes such as* p53* in the incidence of some cancers as well as changes in the incidence of the cell cycle regulator protein and cellular proliferation marker Ki-67 as well as the expression of *HER-2/neu* and *CD44* genes in many cancers, the relationship between their immunohistochemistry, clinical, and pathological indicators of tumors can be determined. Thus, this study aimed to determine the relationship between immunohistochemistry of *p53, CD44, Ki-67*, and *HER-2/neu* genes and demographic characteristics as well as pathological findings, alongside the state and site of cancer and prognosis of these patients to identify high-risk patients and determine the therapeutic regime.

## Materials and Methods

*Methods*

This descriptive-analytical and cross-sectional study was conducted across a population with gastric cancer who underwent gastrectomy surgery in Shohahadey Tajrish Hospital in 2011-2016. The studied population was chosen through simple available sampling in a non-probabilistic way. In order to prepare the samples related to stomach carcinoma, 60 patients who underwent gastrectomy in 2011-2016 were extracted by referring to the archive of the pathology ward of the mentioned hospital, out of the list of gastric cancers. Then, by investigating the relevant slides, the best paraffin block with each having the maximum tumor volume was chosen and separated. After cutting the relevant blocks, hematoxylin and Eosin (H and E) staining and staining through immunohistochemistry (IHC) were performed for* p53*,* CD44, Ki-67*, and *HER-2/neu* markers. The results obtained from the staining of the markers through IHC were evaluated with histopathological findings obtained from the file of patients in the archive and staining (H and E), including age, gender, tumor size, site of tumor (cardia, fundus, antrum), macroscopic view (ulcerative, fungal-shaped, infiltrative), histological degree (G1, G2, and G3), type of tumor according to Loren classification (intestinal, diffuse), T-stage, metastasis to lymph nodes, lymphovascular invasion, and rate of survival.

*Immunohistochemistry and its staining assessment*

The expression of* p53*,* Ki-67*, and *HER-2/neu* was investigated through immunohistochemistry method. Streptavidin, biotin, peroxidase complex method (labeled Strept - Avidin Biotio/HRP) was implemented on 4-mcm cuts of the tissues cut over paraffin blocks. The used slides were hyalinized for better adhesion of the tissue to the glass slide. After cutting and de-paraffinizing in xylol and dewatering in alcohol, the cuts were incubated in hydrogen peroxide 3% in water for 20 min in order to inhibit the activity of internal peroxidase. Then, for retrieval of antigen, the tissue cuts were placed inside buffer citrate solution (pH=6, 0.01 M) and placed inside in an autoclave at 121°C for 20 min. Next, the cuts were stained using the special antibodies of each marker through the following method: For* p53*, Monoclonal Mouse Anti-Human* p53* Protein Clone DO-7 DAKO (Code N1581) and for Ki-67, Monoclonal Mouse Anti-Human Ki-67 Antigen Clone MIB-1 DAKO (Code N1633) were used. Based on the recommendation of the brochures of each of the kits, the initial incubation time for these primary antibodies was 25 min at room temperature. After washing with TBS, the slides were incubated at room temperature by a layer of Biotinylated Goat Anti- Rabbit/Mouse immunoglobulins (secondary antibodies) in a phosphate buffered saline (PBS) containing stabilizing protein. For each of the above-mentioned markers, an extra cut was stained without applying the primary antibody as negative control. Next, streptavidin conjugated with horseradish peroxidase in buffer solution was added to the cuts (incubation time: 25 min). After washing and adding one drop of substrate-Chromogen with 10 min incubation, the slides were prepared for the last stage, which was cross staining with HandE (DAKO LSAB2 System - HRP).

The scoring criteria were considered as follows for *p53, CD44, Ki-67*, and *HER-2/neu* based on the staining intensity and proportion of the nuclear staining. The cutoff value for positivity or negativity of* p53*,* CD44, Ki-67*, and *HER-2/neu* was determined as follows (35):

-* p53* staining was considered positive when any amount of tumor cells was stainable.

- *Ki-67 *staining was considered when >10% of tumoral cells had a medium stainability intensity.

- *HER-2/neu* staining was considered positive when the tumoral cells had moderate (++) staining intensity or above.

- *CD44* staining was considered positive when >50% of the tumoral cells were stainable.

Concerning the ethical principles and confidentiality, information obtained from all samples remained confidential. Moreover, Kolmogorov-Smirnov test was used to examine the distribution normality of the scores. As this test was insignificant, independent t-test was used for comparing two groups, plus Chi-do as well as repeated measures ANOVA. In addition, P-value<0.05 was considered as the minimum value for statistical significance. Finally, all analyses were performed in SPSS 22.

**Figure 1:**
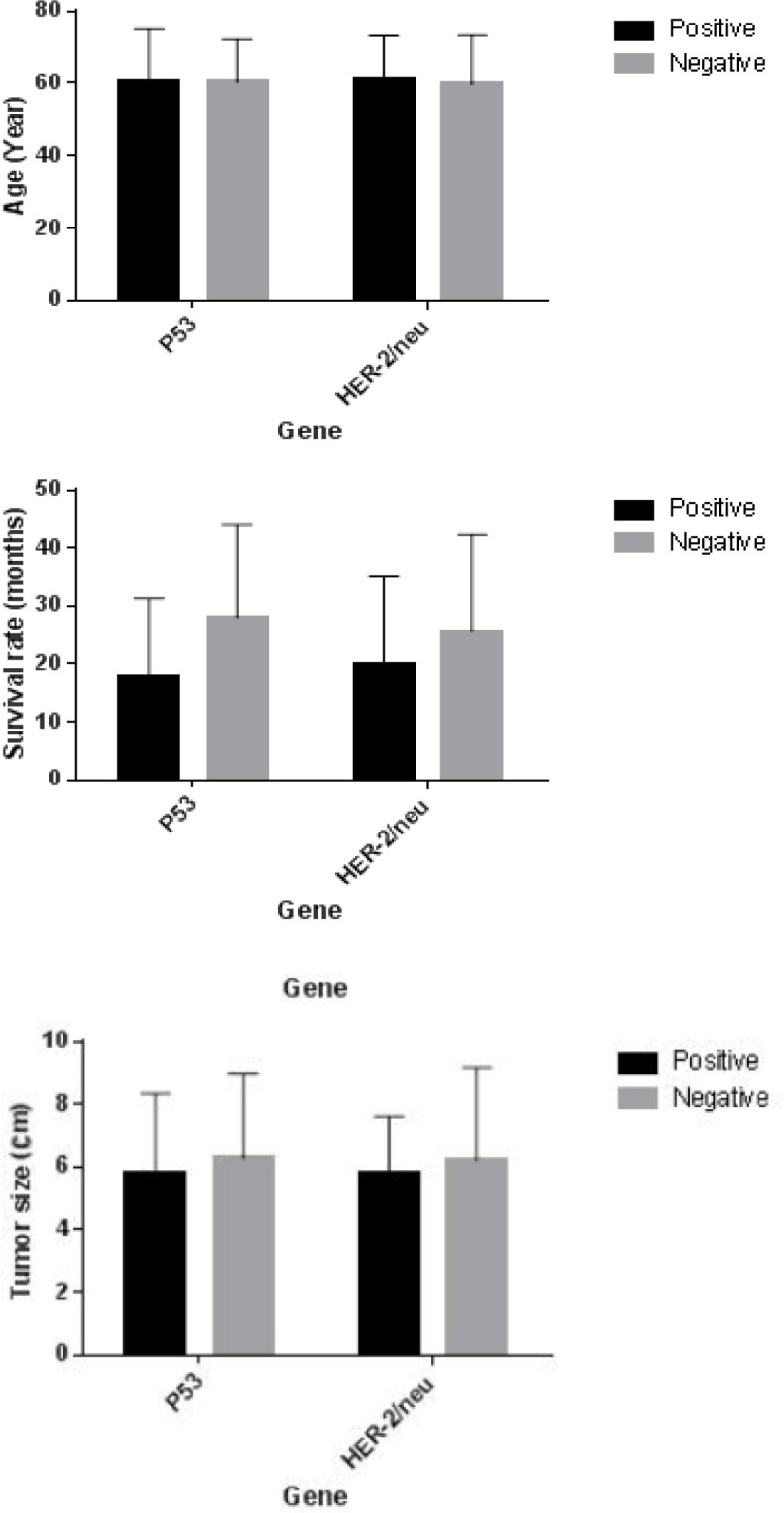
Evaluation of Age, Survival Rate and Tumor Size with Respect to Expression Levels of* P53* and *HER-2/neu*

**Table 1:** Demographic Characteristics of Patients and Their Tumor Properties

Variables	Group	Mean/ Frequency	Stdev/ Percentage
Tumor type	Intestinal	40	66.7
	Diffuse	10	16.7
	Mixed	3	5
	Mucinous	7	11.7
Macroscopic view	Ulcerative	35	58.3
	Polypoid	3	5
	Infiltrative	22	36.7
Tumor location	Corpus	20	33.3
	Fundus	3	5
	Antrum	20	33.3
	Cardia	12	20
	Pre-pyloric	5	8.3
Tumor Grade	Grade 1	6	10
	Grade 2	24	40
	Grade 3	30	50
Tumor Stage	T1	1	1.7
	T2	10	16.7
	T3	20	33.3
	T4	29	48.3
Tumor size (Cm)	-	6.09	2.61
Survival rate (months)	-	23.82	1.56
Ki-67 expression (%)	-	33.75	34.1
CD44 expression (%)	-	24.5	32.2
Lymph node involvement	N0	13	21.7
	N1	14	23.3
	N2	15	25
	N3	18	30
Lymphovascular invasion	Lymph+ Vasc+	28	46.7
	Lymph- Vasc-	17	28.3
	Lymph+ Vasc-	11	18.3
	Lymph- Vasc+	4	6.7
P53 expression	Positive	25	41.7
	Negative	34	56.7
HER-2 / neu expression	Positive	20	33.3
	Negative	40	66.7

**Table 2:** Relationship between Demographic Variables as Well as Tumor Features and the Extent of Expression of* p53* and HER-2/neu

Variables	Group	P53			Her-2/neu		
		Positive	Negative	*P*-Value	Positive	Negative	*P*-Value
Gender	Male	21 (47.7)	22 (50)	0.211	17 (38.6)	27 (61.4)	0.148
	Female	4 (25)	12 (75)		3 (18.8)	13 (81.2)	
Tumor type	Intestinal	19 (76)	20 (58.8)	0.431	19 (95)	21 (52.5)	0.01
	Diffuse	5 (20)	5 (14.7)		0 (0)	10 (25)	
	Mixed	0 (0)	3 (8.8)		0 (0)	3 (7.5)	
	Mucinous	1 (4)	6 (17.6)		1 (5)	6 (15)	
Macroscopic view	Ulcerative	19 (76)	15 (44.1)	0.143	15 (75)	20 (50)	0.034
	Polypoid	1 (4)	2 (5.9)		2 (10)	1 (2.5)	
	Infiltrative	5 (20)	17 (50)		3 (15)	19 (47.5)	
Tumor location	Corpus	9 (36)	10 (29.4)	0.141	7 (35)	13 (32.5)	0.676
	Fundus	0 (0)	3 (8.8)		0 (0)	3 (7.5)	
	Antrum	7 (28)	13 (38.2)		8 (40)	12 (30)	
	Cardia	4 (16)	8 (23.5)		4 (20)	8 (20)	
	Prepyloric	5 (20)	0 (0)		1 (5)	4 (10)	
Tumor Grade	Grade 1	2 (8)	3 (9)	0.118	2 (10)	4 (10)	0.329
	Grade 2	9 (36)	15 (44)		7 (35)	17 (43)	
	Grade 3	14 (56)	16 (47)		11 (55)	19 (47)	
Tumor Stage	T1	0 (0)	1 (2.9)	0.689	0 (0)	1 (2.5)	0.241
	T2	3 (12)	7 (20.6)		1 (5)	9 (22.5)	
	T3	11 (44)	9 (26.5)		9 (45)	11 (27.5)	
	T4	11 (44)	17 (50)		10 (50)	19 (47.5)	
Lymph node involvement	N0	4 (16)	9 (26.5)	0.728	3 (15)	10 (21.7)	0.629
	N1	7 (28)	7 (20.6)		6 (30)	8 (23.3)	
	N2	6 (24)	9 (26.5)		6 (30)	9 (25)	
	N3	8 (32)	9 (26.5)		5 (25)	13 (30)	
Lymphovascular invasion	Lymph+ Vasc+	15 (60)	12 (35.3)	0.556	13 (65)	15 (37.5)	0.166
	Lymph- Vasc-	5 (20)	12 (35.3)		4 (20)	13 (32.5)	
	Lymph+ Vasc-	4 (16)	7 (20.6)		3 (15)	8 (20)	
	Lymph- Vasc+	1 (4)	3 (8.8)		0 (0)	4 (10)	

**Table 3:** Relationship between Demographic Variables as Well as Tumor Features and the Extent of Expression of Ki67 and HER-2/neu

Variables	Group	Ki-67			CD44		
		Mean	Stdev	*P*-Value	Mean	Stdv	*P*-Value
Tumor type	Intestinal	33.36	32.62	0.288	21.5	26.82	0.934
	Diffuse	42.00	39.94		32	41.40	
	Mixed	53.33	47.26		26.67	37.86	
	Mucinous	14.29	25.07		30	47.96	
Gross Appearance	Ulcerative	37.14	35.03	0.654	28.71	32.25	0.447
	Polypoid	16.67	20.82		8.33	10.41	
	Infiltrative	30.68	34.17		20	33.52	
Tumor location	Corpus	30.00	33.72	0.704	15	21.64	0.435
	Fundus	13.33	23.9		6.67	11.55	
	Antrum	38.5	32.97		35	37.17	
	Cardia	37.08	35.70		24.17	32.88	
	Prepyloric	34.00	47.75		32	46.04	
Tumor Grade	Grade 1	26.67	28.05	0.389	22.5	27.88	0.631
	Grade 2	40.63	33.11		26.25	28.41	
	Grade 3	29.67	35.96		23.5	36.42	
Tumor Stage	T1	0.00		0.732	0		0.64
	T2	38.00	39.67		34	42.22	
	T3	35.00	34.72		26.5	31.79	
	T4	32.59	32.8		20.69	29.15	
Lymph node involvement	N0	31.54	39.34	0.786	24.62	37.33	0.957
	N1	31.43	30.35		24.64	25.61	
	N2	41.67	38.25		24.67	29.24	
	N3	30.56	30.96		24.17	37.43	
Lymphovascular involvement	Lymph+ Vasc+	38.39	32.06	0.55	28.04	32.56	0.615
	Lymph- Vasc-	24.71	33.75		21.18	38.55	
	Lymph+ Vasc-	32.73	36.08		22.27	24.04	
	Lymph- Vasc+	42.50	49.24		20	32.16	

**Figure 2:**
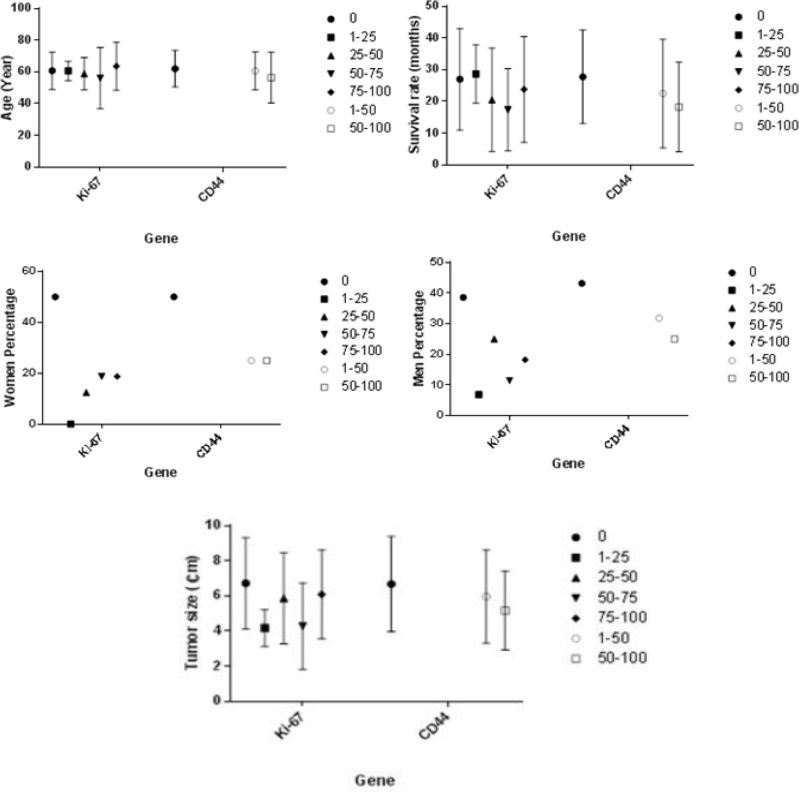
Evaluation of Age, Gender, Survival Rate and Tumor Size with Respect to Expression Levels of Ki-67 and CD44

**Figure 3:**
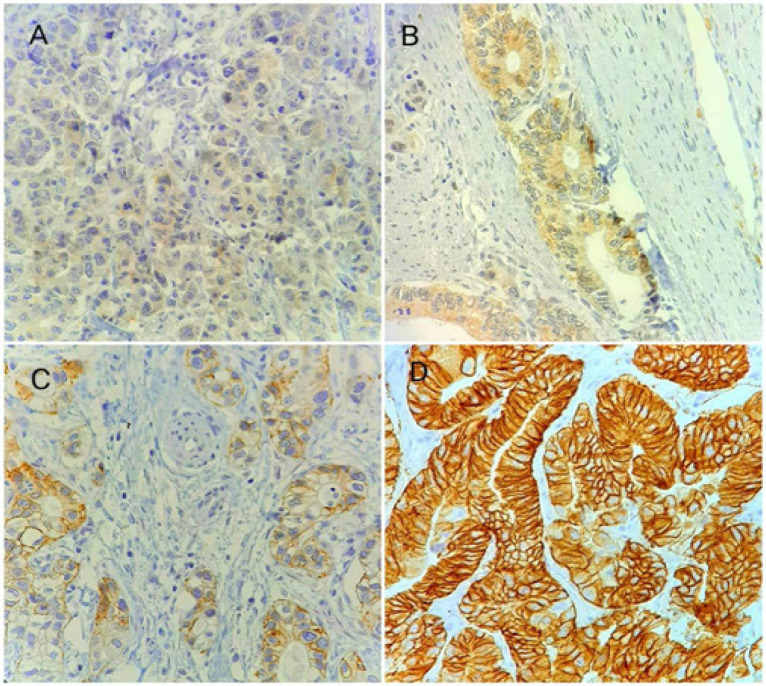
HER2/neu Expression in Gastric Adenocarcinoma. A, score 0; B, score 1+; C, score 2+ and D: score 3+.

**Figure 4:**
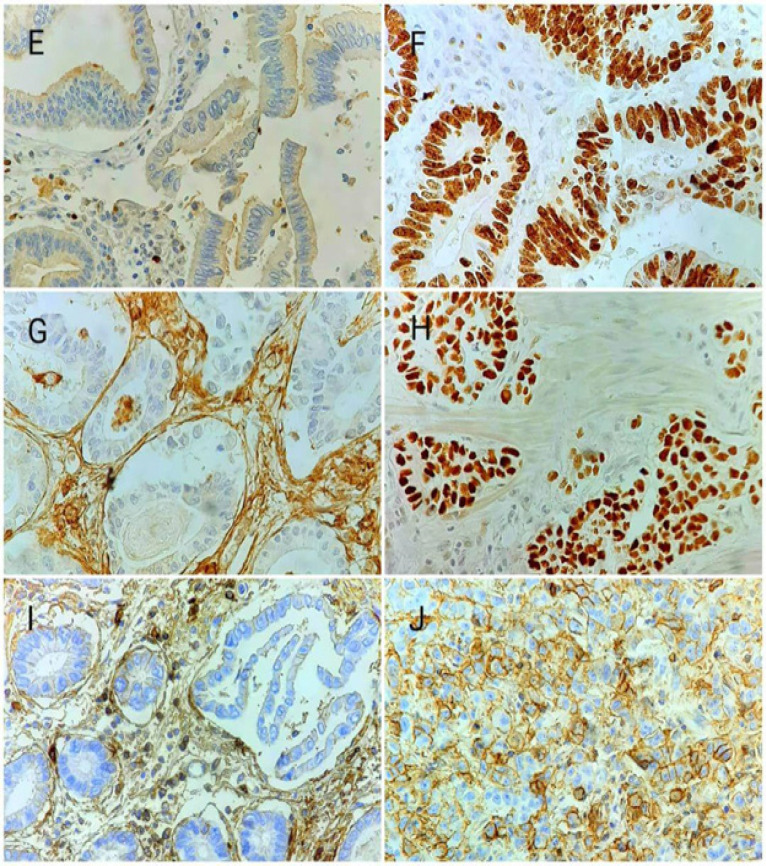
*Ki-67, P53, CD44* Expression in Gastric Adenocarcinoma. (E), Negative and (F) Positive Ki-67; (G), Negative and (H) Positive* P53*; (I), Negative and (J) Positive CD44

## Results

Demographic characteristics of patients and their tumor properties were investigated in this study. Out of the 60 patients studied, 44 were male and 16 were female. The mean age of the patients was 60.25±1.29, and the extent of the patients’ survival was 23.82±1.56 months on average. Table 1 reports other results obtained from this investigation.

Based on the investigations in this study, the intended variables, including the tumor features and information of patients were investigated considering the extent of expression of* p53* and *HER-2/neu*. Table 2 shows the results obtained from the relationship between demographic variables as well as tumor features and the extent of expression of* p53* and *HER-2/neu*. According to the results, the rate of the patients’ survival had a significant relationship with the incidence of* p53* (p-value=0.014). This means that the expression of* p53* gene was associated with a lower rate of survival in the stomach adenocarcinoma patients. Investigating *HER-2/neu*, as with *p53*, in the patients with this marker demonstrated that 75% had an ulcerative tumor, and 50% and 47.5% in negative *HER-2/neu *patients had ulcerative and infiltrative tumors, respectively. This difference was significant (p=0.034), and the expression of *HER-2/neu* was associated with ulcerative tumors with a higher probability. Moreover, according to the results, there was a significant relationship between the type of tumor and the expression of *HER-2/neu* with p=0.010. Based on this finding, it could be stated that the expression of *HER-2/neu *gene was more likely to be associated with the intestinal type of stomach adenocarcinoma.

The present study investigated demographic characteristics of the patients including age, gender, and rate of survival based on the extent of expression of *Ki-67* and *CD44* according to stainability. Diagram 1 shows the results. In addition, the tumor characteristics were investigated considering the extent of expression of *Ki-67* and *CD44*. Table 3 presents the results obtained from the relationship between tumor characteristics and the extent of expression of *Ki-67* and *CD44*. According to the results mentioned in Diagram 1 and Table 3, no significant relationship was found between demographic characteristics and tumor features considering the extent of expression of *Ki-67* and *CD44*.

## Discussion

This study was a descriptive-analytical study in which the stomach file of 60 patients with cancer undergoing gastrectomy in Tajrish Hospital in 2011-2016 was extracted and investigated for staining *p53, Ki-67, CD44*, and *HER-2/neu*, along with the demographic characteristics of patients, rate of survival, and histopathological features of tumors. Based on the findings of the study,* p53* and *CD44* markers do not have any diagnostic value in predicting the biological behavior of gastric cancer. Moreover, incidence of* p53* mutation expression was related to the lower rate of survival, and the expression of *HER-2/neu* was related to higher probability of developing the intestinal type of stomach adenocarcinoma and ulcerative macroscopic view (Shafigh et al., 2007). 

In the study by Staib et al., (2005), it was found that chronic inflammation can initiate carcinogenesis and cause progress of tumor and metastasis. Indeed, inflammatory response is a complicated biological network, and* p53* plays a key role in its expression. Therefore, patients with* p53* mutations have a worse prognosis compared to the patients without this mutation. The study conducted by Pinto-de-Sousa et al., (2004) on 163 patients with cancer showed that mutation in* p53* gene was associated with the vascular invasion, lymphatic metastasis, and higher five-year survival, and the expression of this gene was associated with more aggressive characteristics in the stomach carcinoma. The present study also showed that the patients with a mutation in* p53* had a significantly lower rate of survival (around 11 months). In this population of patients, larger percentages of lymphatic meta-stasis and lymphovascular invasion were observed in comparison with the patients without it (though not statistically significant). Moreover, Joypaul et al., (1994) investigated a sample of 260 patients with stomach adenocarcinoma and revealed that* p53* was positive in 46% patients (in our study, 41.7% of patients were* p53* positive), and similar to the present study, there was no significant relationship between the expression of this marker and grade of tumor, type of tumor (based on the Lauren classification), and lymphatic metastasis; however, a significant relationship existed only between the expression of* p53* and the survival rate of patients (with odds ratio = 1.89, 95% confidence interval 1.33-2.69). For the death and five-year survival, it was 3% in patients with* p53* expression compared to 16% in patients without it. Their findings were similar to the results of the present research.

The expression of HER-2/neu has been proved to occur in stomach adenocarcinoma and the gastroesophageal junction. In spite of the high probability of association, the expression of this marker is related to poorer prognosis, resistance to conventional chemotherapy in this malignancy, and other carcinomas. Recently, researchers found that when this marker is expressed, anti-HER2 antibody trastuzumab treatments can be used, and thus its detection has a high therapeutic value (Hechtman and Polydorides, 2012). Retrospective investigation of 120 patients with gastric cancer following surgery by Una Cidon (2012) indicated that the expression of HER-2/neu gene was observed in 7.5% of patients and its positivity was significantly correlated with female gender, intestinal form, and more advanced stages of this cancer. According to the study by Park et al., (2006) on 180 patients with gastric cancer, 15.9% of patients, in which HER-2/neu had been expressed, highly experienced the intestinal type of tumor, and these patients had a shorter five-year survival compared to those without it. However, this gene expression in the present study was higher (33.3%) and its expression, as with this study, was significantly related to the intestinal form of adenocarcinoma. On the other hand, in spite of the lower rate of survival in positive HER-2/neu patients (20.15 months versus 26.65 months), this difference was not significant. In several other studies, the expression of HER-2/neu was associated with the intestinal type of adenocarcinoma and poorer prognosis (Liu et al., 2012; Hsu et al., 2011; Wang and Zhang 2017). Based on the previous studies, membrane immunoactivity is observed only in the intestinal type (Lemoine et al., 1991; Falck and Gullick 1989). However, the reasons for the selective expression of HER-2/neu in the intestinal type of carcinoma seems to be complex. This oncogene is associated with the intestinal phenotype of carcinoma. Nevertheless, since not all intestinal type tumors show the expression of HER-2/neu, it cannot be the only factor involved in the development of such a type of adenocarcinoma. In addition, it seems unlikely that all intestinal tumors be developed by an external cause, and this may justify the different prevalence of the expression of HER-2/neu across different cases. Finally, investigation of 1036 patients by Hsu et al., (2011) and based on the meta-analysis by Wang and Zang (2017), it was found that there is a relationship between the expression of this marker and the differentiated phenotype.

Based on the research, Ki-67 antigen plays a significant role in regulating cellular proliferation and has prognostic value in many types of cancer (Huang et al., 2016). In the present study, Ki-67, as an independent factor, had no diagnostic value in predicting the biological behavior of stomach adenocarcinoma and rate of patients’ survival. Huang et al., (2016) study demonstrated that high Ki-67 expression (immunohistochemistry score > or = 3+) was a significant prognostic factor for relapse, remote metastasis, and overall survival of patients. The study conducted by Stahl et al., (2017) also showed that the expression of this biomarker was associated with shorter survival. The reason for the different result may be because of differences in immunohistochemical staining and scoring systems. Finally, Shafigh et al., (2007) study on 58 patients revealed that investigation of* p53* and Ki-67 markers alone had no diagnostic value in predicting the biological behavior of gastric cancer.

According to the studies, CD44 is one of the most common markers of cancer stem cells (CSCs) in gastric cancer, which has the potential for regeneration, initiation of tumor progression, and possibly development of metastasis. In the present study, no significant relationship was found between the extent of expression of this biomarker and demographic characteristics or histological features of patients. Moreover, (Wakamatsu et al., 2012; Lu et al., 2016; Ibrahim et al., 2018) concluded that the expression of CD44 is associated with a poorer prognosis and lower survival of patients (Wakamatsu et al., 2012; Lu et al., 2016; Ibrahim et al., 2018). The result difference may be due to methodology and/or sample size. In addition, Nosrati et al., (2014) investigated 95 samples of the primary adenocarcinoma of the stomach and observed a relationship between CD44 and the intestinal type of tumor, tumor size (4-8 cm), depth of invasion, and moderate differentiation.

Of course, some studies investigated these four biomarkers concurrently. In a study conducted by Sanat et al., (2017) on 100 patients, it was found that only* p53* and *CD44 *were associated with the lifespan of patients, and no relationship was found between the expression of *Ki-67* or *HER-2/neu* and the extent of survival of patients.

In conclusion, according to the results,* p53* and *CD44* markers had no diagnostic value in predicting the biological behavior of the gastric cancer. Moreover, incidence of mutation in* p53* gene was related with the lower rate of survival, and the expression of *HER-2/neu* was associated with higher probability of developing the intestinal type of stomach adenocarcinoma and ulcerative gross feature. Therefore, it was suggested that future studies address a larger sample size in this regard, matching the sample as to gender, types ratio and grades. In addition, future studies can deal with the comparison of other markers such as other CSCs, including CD133, examination of the relapse of patients following surgery, and the relationship between markers and their effect on the relapse of adenocarcinoma.
